# Selective Venous Catheterization for the Localization of Phosphaturic Mesenchymal Tumors

**DOI:** 10.1002/jbmr.316

**Published:** 2010-12-16

**Authors:** Panagiota Andreopoulou, Claudia E Dumitrescu, Marilyn H Kelly, Beth A Brillante, Carolee M Cutler Peck, Felasfa M Wodajo, Richard Chang, Michael T Collins

**Affiliations:** 1 Skeletal Clinical Studies Unit, Craniofacial and Skeletal Diseases Branch, National Institute of Dental and Craniofacial Research, National Institutes of HealthBethesda, MD, USA; 2 Diagnostic Radiology Department, National Institutes of HealthBethesda, MD, USA; 3 Musculoskeletal Oncology, Inova Fairfax HospitalFairfax, VA, USA

**Keywords:** FGF-23, Osteomalacia, Rickets, Tumor-Induced Osteomalacia, Oncogenic Osteomalacia

## Abstract

Tumor-induced osteomalacia (TIO) is characterized by renal phosphate wasting, hypophosphatemia, and aberrant vitamin D_3_ metabolism and is caused by fibroblast growth factor 23 (FGF-23)–producing mesenchymal tumors, which are often difficult to locate. We investigated the utility of selective venous sampling in tumor localization. The primary endpoint was identification of the FGF-23 concentration ratio between the venous drainage of the tumor bed and the general circulation that was diagnostic of the location of an FGF-23-secreting tumor. Fourteen subjects underwent 15 sampling procedures after functional and anatomic imaging studies. Subjects fit into three imaging categories: no suspicious site, multiple sites, and single site (positive controls). FGF-23 levels were measured by ELISA. Suspicious tumors were resected for diagnosis, confirmation, and cure. In subjects with a positive venous sampling study and subsequent cure, a minimum ratio of 1.6 was diagnostic. In 7 of 14 subjects there was suggestive imaging, a diagnostic ratio, and an associated TIO tumor (true positive). Four of these required complicated resection procedures. In 4 of 14 subjects with no suspicious site on imaging studies, an FGF-23 diagnostic ratio was not detected (true negative). Biopsy or resection of a single lesion in 2 of 14 subjects with a diagnostic ratio failed to identify a TIO tumor (false positive). A diagnostic FGF-23 ratio was absent in 1 of 14 subjects whose tumor was a single highly suspicious lesion on imaging studies (false negative). These data yield a sensitivity of 0.87 [95% confidence interval (CI) 0.47–0.99] and a specificity of 0.71 (95% CI 0.29–0.96). Selective venous sampling for FGF-23 was particularly useful in subjects with multiple suspicious sites or an anatomically challenging planned resection but not in the absence of a suspicious lesion on imaging studies. © 2011 American Society for Bone and Mineral Research.

## Introduction

Osteomalacia is a metabolic bone disorder characterized by defective mineralization, reduced formation of mature bone, and the accumulation of osteoid. Tumor-induced osteomalacia (TIO), also known as *oncogenic osteomalacia*, is a rare acquired paraneoplastic disorder characterized by hypophosphatemia, renal phosphate wasting, hyperphosphaturia, osteomalacia, and inappropriately low serum 1,25-dihydroxyvitamin D_3_ [1,25(OH)_2_D_3_] levels.(1,2) Clinical features include progressive bone pain, muscle weakness, fatigue, gait disturbances, and recurrent fractures. TIO is usually caused by phosphaturic mesenchymal tumors (PMTs) of mixed connective tissue variant type (MCT) that produce and secrete fibroblast growth factor 23 (FGF-23).(3,4) FGF-23 is a 32-kDa protein identified in 2000 as the protein mutated in autosomal dominant hypophosphatemic rickets (ADHR)(5–7) and recognized as an important regulator of phosphate and vitamin D metabolism. It consists of an N-terminal region containing the FGF homology domain and a 71-amino-acid C-terminus. Data thus far support the model that FGF-23 binds to Klotho and an FGF receptor in the renal tubule and inhibits both phosphate reabsorption via action on the sodium-phosphate cotransporters 2a (NaPi-IIa) and 2c (NaPi-IIc) and 1α-hydroxylation of 25-hydroxyvitamin D [25(OH)D] to 1,25(OH)_2_D_3_. It also stimulates the 24-hydroxylase that inactivates 1,25(OH)_2_D_3_ at the proximal renal tubule. The likely primary physiologic source of circulating FGF-23 is bone cells, especially osteocytes.(8–11)

To date, there have been a few case reports on the successful use of selective venous catheterization and sampling for the localization of PMTs.(12–16) As this technique becomes more widely used, it is important to identify criteria for a diagnostic study and the circumstances under which the procedure is likely to be of clinical utility.

The objectives of this study were the identification of an FGF-23 concentration ratio between the venous drainage of the tumor bed and the general circulation that was diagnostic of the location of an FGF-23-secreting tumor and to assess the utility of selective venous sampling for FGF-23 measurement in tumor localization in subjects with either negative or inconclusive imaging studies.

## Subjects, Materials, and Methods

### Study subjects

The protocol was approved by the institutional review board, and informed consent was obtained from all subjects or their guardians. The diagnosis of TIO was confirmed by a typical clinical history, absence of family history suggestive of a hereditary hypophosphatemic disorder, and biochemical confirmation based on hypophosphatemia, renal phosphate wasting, low or inappropriately normal serum 1,25(OH_2_)D_3_ level, and an elevated serum intact FGF-23 level. Fourteen subjects (9 males and 5 females) between 16 and 63 years of age (median 47 years, mean 45.5 years) underwent 15 venous sampling procedures. One subject underwent 2 venous sampling procedures. The subjects' baseline characteristics are shown in Table 1.

**Table 1:** Baseline Characteristics

Subject	Age (years)	Serum P (mg/dL)	TmP/GFR	FGF-23 (pg/mL)^a^	1,25(OH)_2_D_3_ (pg/mL)	iPTH (pg/mL)	Treatment^b^

Normal range	NA	2.5–4.8 mg/dL	2.7–4.5	10–50 pg/mL	22–67 pg/mL	16–87 pg/mL	NA
1	35	2.3	1.97	64	41	30.3	Y
2	47	1.8	0.71	355	<10	10.8	Y
3	61	2.2	1.88	606^c^	<10	32.7	Y
4	50	1.6	1.24	1684	<10	15.7	N
5	57	2.1	1.05	473	19	63.9	Y
6	40	1.8	1.39	1113	<10	30.2	N
7	55	1.2	0.78	406	62	98.6	Y
8	45	1.6	1.45	353	11	29.8	N
9	33	2.2	1.46	146	32	87.1	Y
10	16	1.6	0.99	68	46	26.9	N
10	16	1.6	0.99	68	46	26.9	N
11	37	1.8	0.93	290	13	50.8	N
12	51	2.6	1.57	472	29	81.5	Y
13	47	1.9	0.92	1029	43	54.1	Y
14	63	2.0	1.54	156	12	121	N

P = phosphate; TmP/GFR = renal tubular reabsorption of phosphate per glomerular filtration rate; FGF-23 = fibroblast growth factor 23; 1,25(OH)_2_D_3_ = 1,25-dihydroxyvitamin D_3_; iPTH = intact parathyroid hormone; NA = not applicable.

a Kainos assay, Japan.

b Treatment consisted of calcitriol and phosphate supplementation.

c Immutopics assay, San Clemente, CA, USA.

None of these subjects in whom no definitive tumor site was identified had a family history suggestive of a possible genetic etiology of the hypophosphatemia. Subject 1 presented with recurrent disease after the initial identification and resection of a PMT in the right maxilla but with tumor present at the surgical margins. Given the initial confirmed diagnosis of TIO, genetic testing was not indicated. Subject 3 had a negative FGF-23 genetic analysis (for ADHR). Because he presented at age 56, a *PHEX* mutation was not tested. Subject 4 did not undergo genetic testing because he also presented at age 50. Subject 9 had a negative FGF-23 genetic analysis and rather late presentation at age 33. Subject 10, who presented in childhood at around age 12, had both PHEX and FGF-23 genetic analyses that were negative. None of these subjects underwent *DMP1* mutation testing because there was no family history of consanguinity or suggestive clinical picture.

### Biochemical evaluation

Biochemical testing consisted of a serum creatinine, inorganic phosphate level, intact parathyroid hormone (iPTH), 1,25[OH]_2_D_3_ level, 25(OH)D level, second-void morning urine phosphate and creatinine, and baseline serum intact FGF-23 level.

### Imaging evaluation

After confirmation of the diagnosis of TIO, all patients underwent functional imaging consisting of indium-111 octreotide (pentetreotide) scintigraphy, [^18^F]fluorodeoxyglucose positron emission tomography with computed tomography (FDG-PET/CT), and whole-body technetium-99 sestamibi scanning. Locations with increased tracer uptake were investigated further with anatomic imaging that included radiographs, computed tomography (CT), and/or magnetic resonance imaging (MRI).

### Selective venous sampling

Subjects with negative or inconclusive imaging findings underwent venous catheterization and sampling under fluoroscopic guidance for FGF-23 measurements by the same experienced interventional radiologist (RC) at the Mark O Hatfield Clinical Center at the National Institutes of Health Clinical Center. The catheter was inserted through the femoral or internal jugular vein. Seventeen major veins and their branches were sampled. Between twenty-five and forty-nine 1.5- to 2-mL samples per subject (mean 31 samples) were taken. Five subjects with a highly suspicious tumor location on imaging were included as positive controls and underwent more focused sampling from the veins draining the suspected tumor bed. Serum intact FGF-23 level was measured in previously unthawed samples that were stored at −80°C by ELISA (Kainos, Tokyo, Japan) per the manufacturer's instructions. This assay has a sensitivity of 3 pg/mL. The intra- and interassay coefficients of variation are 3% or less and 4% or less, respectively.

Suspicious tumors were resected for final diagnosis, confirmation, and cure. One subject (number 14) who had an intracranial lesion opted not to have surgery and underwent focused irradiation.

### Primary endpoint

The primary endpoint was identification of an FGF-23 concentration ratio between the tumor bed venous drainage and the circulation that was diagnostic of an FGF-23-secreting tumor. The diagnostic ratio was determined by evaluating only studies in patients who were surgically cured and was derived by dividing the maximum FGF-23 value by the mean of the values that represented the general circulation.

### Statistical analysis

The assumption was that when a tumor was present (positive study), there would be one or several FGF-23 values that were significantly higher than the others and that if a subject's FGF-23 values followed a normal distribution, it would likely represent a negative study. Whether or not a set of values was normally distributed was assessed by several tests of normality [ie, Shapiro-Wilk, Kolmogorov-Smirnov (K-S), Cramér-von Mises, and Anderson-Darling]. However, it is possible that a set of FGF-23 values that was normally distributed still could represent a positive study. If this were the case, the highest values would be anatomically contiguous. For this reason, in studies in which the data followed a normal distribution, the highest values also were assessed for anatomic distribution, and if the highest values were noncontiguous, it was concluded that it was a negative study. In all studies with a normal distribution, this was the case.

For positive studies, the maximum FGF-23 value was divided by the mean of the values that represented the general circulation. The FGF-23 concentration that represented the general circulation was determined by calculating the mean of all the remaining values after excluding the maximum FGF-23 value and any other values that were anatomically contiguous with the site from which the maximum value was measured and by qualitative inspection also were elevated.

## Results

### Study population

During the initial screening, eight subjects were receiving conventional treatment with phosphate and/or calcitriol supplementation, and six subjects were not receiving any treatment. All subjects were hypophosphatemic, with serum phosphate levels ranging between 1.6 and 2.3 mg/dL (reference range 2.5 to 4.8 mg/dL), with the exception of one patient under treatment, in whom serum phosphate was 2.6 mg/dL. All subjects had reduced renal tubular reabsorption of phosphorus and elevated baseline intact FGF-23 levels ranging from 64 to 1605 pg/mL (reference range 10 to 50 pg/mL). 1,25(OH)_2_D_3_ levels ranged from undetectable to normal (Table 1).

### Clinical outcome

In 5 subjects, there was a single suspected lesion that, based on both functional and anatomic imaging, was considered highly likely to be the offending tumor. These subjects were studied as positive controls (subjects 2, 5, 6, 7, and 13). In another 5 subjects, based on functional and anatomic imaging, there were multiple possible locations for the tumors (subjects 8, 10, 11, 12, and 14). In 4 subjects, functional and anatomic imaging failed to reveal any suspect lesion (subjects 1, 3, 4, and 9).

Evidence of a clinically significant FGF-23 ratio was present in 4 of the 5 positive control subjects (subjects 2, 5, 6, and 13) and in all 5 subjects with multiple suspect lesions on imaging studies (subjects 8, 10, 11, 12, and 14). FGF-23 levels from all 4 subjects who did not have an identifiable suspect lesion on imaging studies and 1 of the 5 subjects with a highly suspicious single lesion on imaging (subject 7) followed a normal distribution and were considered negative studies. In positive studies, the ratio, as determined by dividing the maximum FGF-23 sample by the mean of the negative values, ranged from 1.6 to 18.9 (Table 2). The differences between the maximum value and the mean (gradient) ranged from 182 to 2753 pg/mL (Table 2).

**Table 2:** Venous Sampling Gradients and Ratios

Subject	Maximum FGF-23 value	Mean FGF-23 value	Gradient	Ratio
2	950	NA^a^	NA	NA
5	569	199	370	2.9
6	4382	1629	2753	2.7
8	485	199	286	2.4
11	433	251	182	1.7
13	1881	1172	709	1.6
14	398	21	377	18.9
Mean	1299	579	779.5	5.0

Ratio determination = highest FGF-23 value/mean FGF23 value of negative samples in pg/mL. See “Subjects, Materials, and Methods” for details.

a Other site values unavailable in intact (Kainos) assay.

**Fig. 1:**
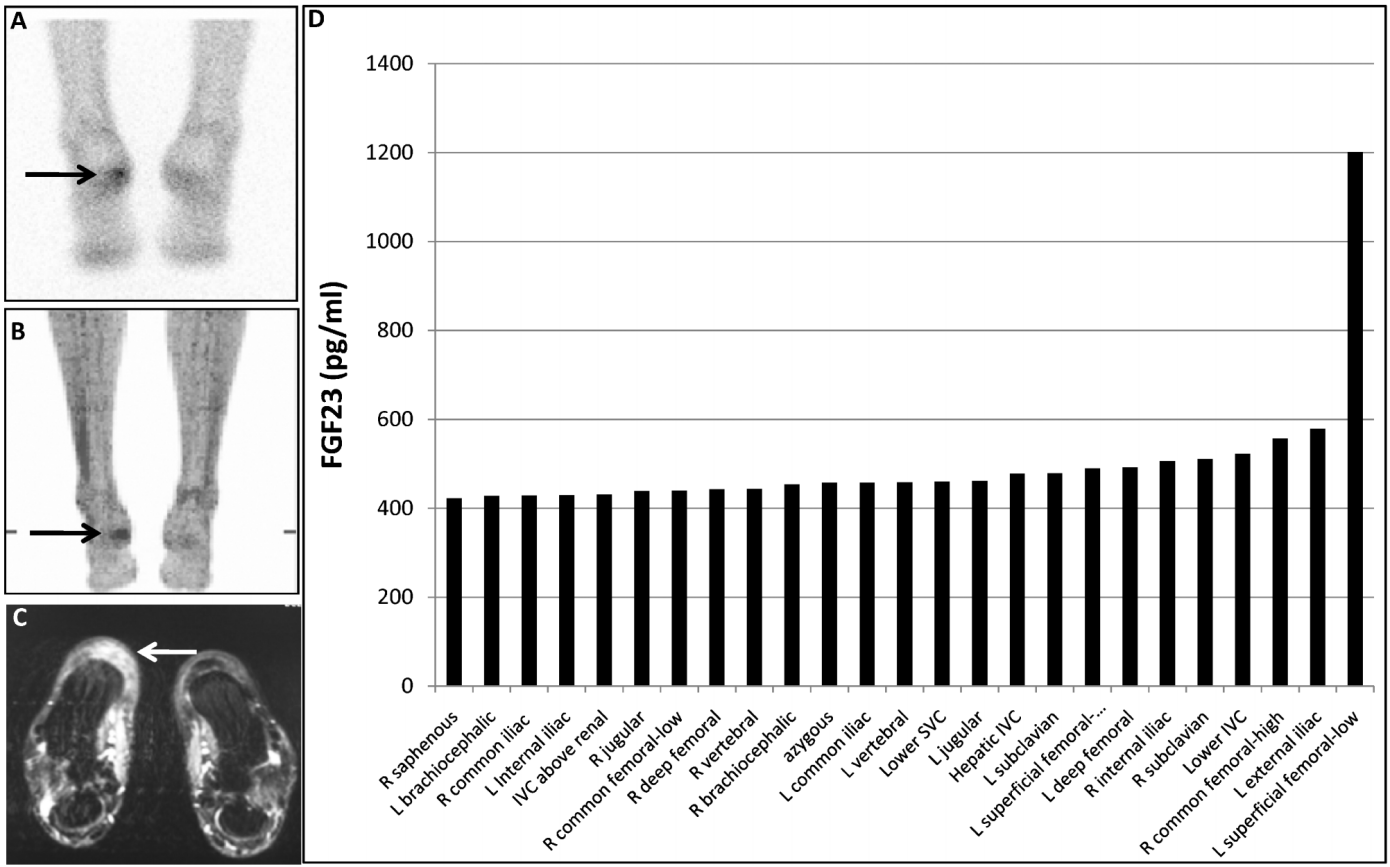
Example of a positive result on selective venous sampling. A suspicious lesion was identified by indium-111 pentetreotide scintigraphy (*A*) and FDG-PET scan (*B*) that correlated with an ill-defined lesion in the fat pad of the heel on MRI (*C*). Surgical removal of the fat pad would entail an extensive procedure that involved the translocation of a vascularized muscle flap from the arm. The results of the selective venous sampling measurements of FGF-23, which confirmed that the lesion was the FGF-23-secreting lesion, are shown (*D*) The tumor is indicated by the arrows.

**Fig. 2:**
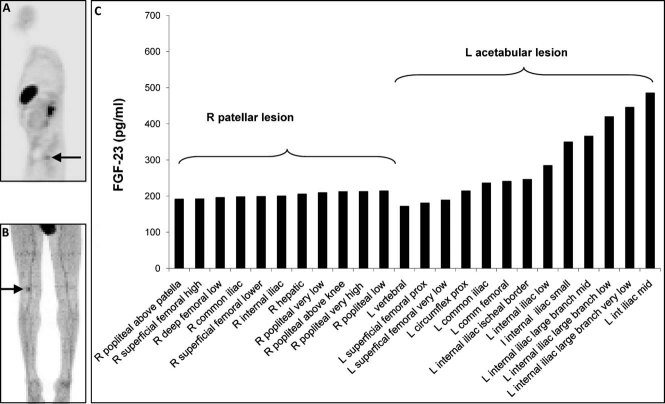
Example of a venous sampling that distinguished between multiple positive images. FDG-PET imaging identified suspicious lesions in both the acetabulum (*A*) and the patella (*B*) that were suspicious for the FGF-23-secreting tumor. The results of the selective venous sampling measurements of FGF-23 (*C*) confirmed that the lesion in the acetabulum was the FGF-23-secreting tumor. The suspicious lesions are indicated by the arrows. This was confirmed by surgical removal and resolution of hypophosphatemia.

**Fig. 3:**
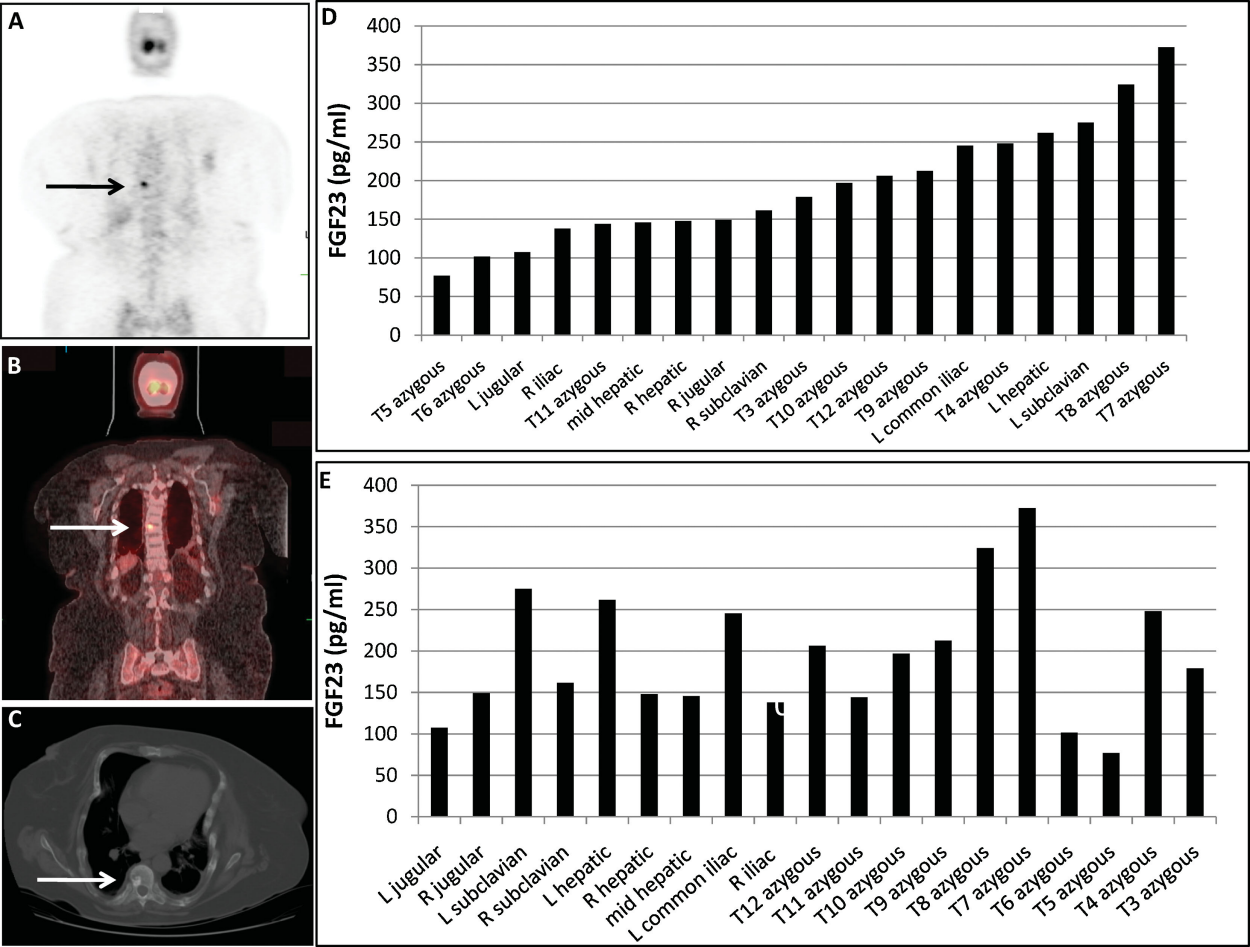
Example of a false-negative selective venous sampling. The pentetreotide scan (*A*), FDG-PET/CT scan (*B*) and CT scan (*C*) identified a lesion in the T_8_ vertebra. However, the results of the selective venous sampling measurements of FGF-23, sorted from lowest to highest (*D*) or anatomically (*E*), failed to identify a confirmatory FGF-23 concentration ratio. Fine-needle aspiration of the lesion and determination of FGF-23 in the aspirate confirmed that the lesion was the FGF-23-secreting tumor. The tumor is indicated by arrows. Surgical excision of the vertebra resulted in cure.(17)

**Fig. 4:**
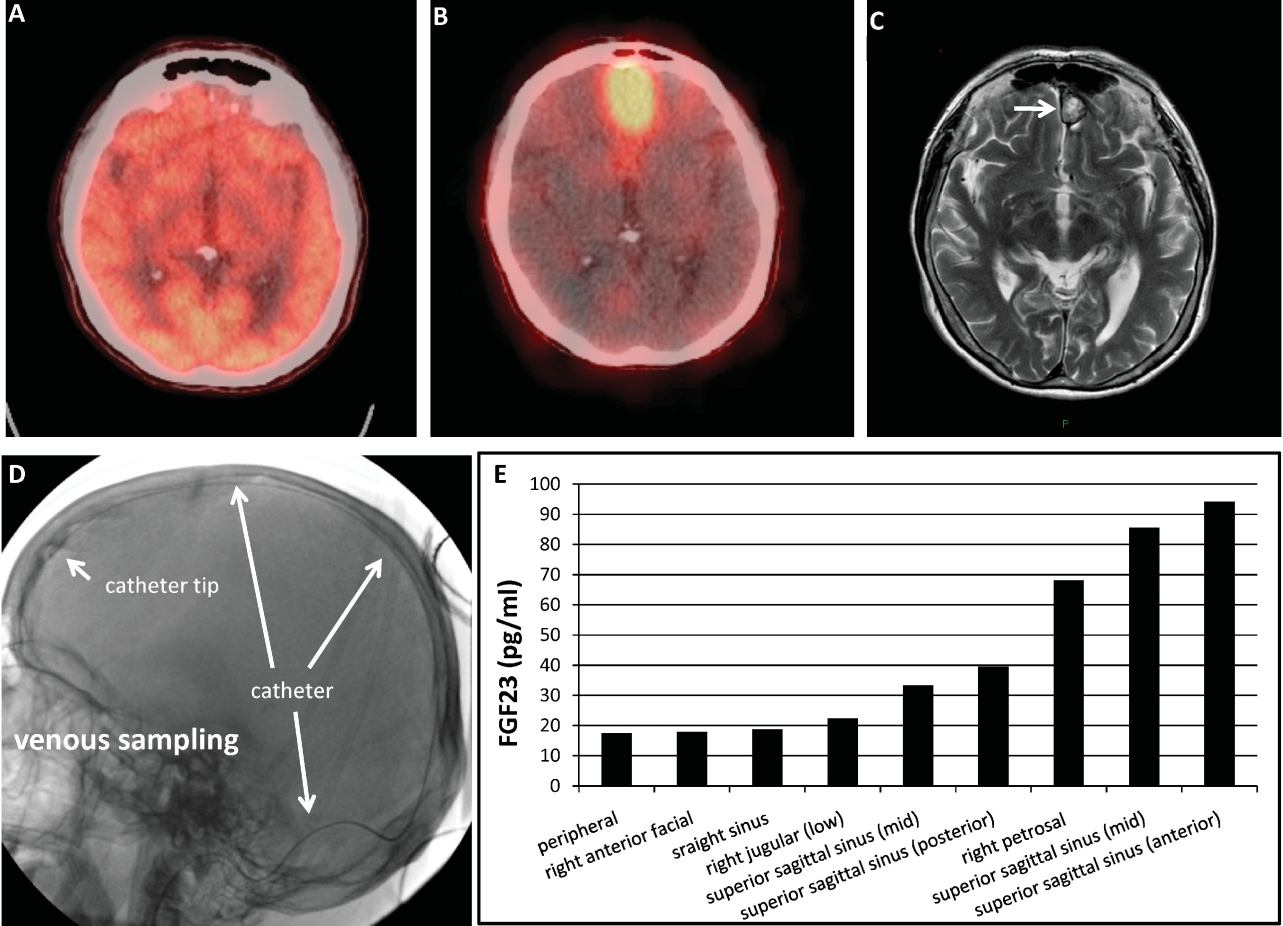
Example of a selective venous sampling that distinguished between conflicting imaging results. For a lesion that ultimately was found adjacent to the brain, FDG-PET/CT scan (*A*) was negative owing to the intense physiologic uptake of FDG by the brain. The pentetreotide/CT scan was positive (*B*), but the MRI findings (*C*) were felt to be most consistent with a meningioma, which also takes up pentetreotide. To resolve the matter, the superior sagittal sinus was catheterized from a transjugular approach (*D*), and FGF-23 concentrations were determined (*E*). The catheter and catheter tip are identified as indicated by white arrows. The results of the venous sampling were consistent with the fact that the lesion identified on pentetreotide scan and MRI was the FGF-23-secreting tumor.

A representative true-positive study in a subject in whom the tumor was identified in the fat pad of the heel of the foot and later resected for cure is shown in Fig. 1. A representative study from a subject with two potentially positive sites on imaging studies (patella and acetabulum) in which venous sampling helped to distinguish a true- from a false-positive imaging study is shown in Fig. 2. The venous sampling results clearly suggested that the acetabular lesion was the culprit, and this was confirmed on resection and normalization of the serum phosphorus level. An example of a false-negative sampling study is shown in Fig. 3. This subject had a clearly identified lesion on functional and anatomic imaging and was included in the positive control group. However, the results of the venous sampling followed a relatively normal distribution, and the sampling results were considered negative. Greater certainty was achieved that the lesion identified in the T_8_ vertebra on imaging studies was the offending tumor by performing CT-guided fine-needle aspiration of the lesion and determining the FGF-23 concentration in the aspirate and the peripheral circulation (aspirate 1140 pg/mL and peripheral circulation 157 pg/mL). Cytology was consistent with a PMT. Resection of the lesion resulted in cure.(17) Subjects 1, 3, 4, and 9, in whom no culprit lesions were found on either imaging or venous sampling, had persistent disease and remain under medical treatment (true negative). All other subjects (2, 5, 6, 8, 10, 11, 12, 13, and 14) had one or multiple suspicious lesions on imaging and underwent resection of a single lesion corresponding to the highest gradient site. Seven of the subjects were cured after resection (2, 5, 6, 8, 11, 13, and 14; true positive). With the exception of an angiolipoma (subject 5), all other tumors were PMTs/MCTs. A biopsy of the target lesion of subject 12 was consistent with an osteoma; thus it was not resected (false positive). Subject 10 underwent two noncurative resections following two venous catheterizations (true negative and false positive). The confirmed tumors were variably located in either soft tissue (ie, heel pad, thigh, and popliteal fossa) or bone (ie, acetabulum, greater trochanter, distal femur, fibular head, and vertebral body; Table 3). In a subject who had conflicting imaging data, venous sampling was able to identify the tumor. In this patient, PET imaging was negative, and pentetreotide scan identified a lesion near the frontal lobe. However, the CT and MRI were felt to be most consistent with a meningioma, which also takes up tracer on pentetreotide imaging. Sampling of the venous drainage of the skull confirmed that the lesion near the frontal lobe was most likely the source of the FGF-23 (Fig. 4). The subject elected not to undergo surgery and opted for focused external-beam irradiation; therefore, the final pathology is not available. However, 6 months after irradiation, the serum phosphorus level is higher on less phosphate supplementation, and the plasma FGF-23 concentration is lower (101 pg/mL from 166 pg/mL before treatment).

**Table 3:** Clinical Outcomes

Subject	Target lesion	Diagnostic ratio	Site	Pathology	Outcome	Comment
1	No	No	None identified	NA	Persistent disease	True negative
2	Yes	Yes	Left heel	PMT	Cured	True positive
3	No	No	None identified	NA	Persistent disease	True negative
4	No	No	None identified	NA	Persistent disease	True negative
5	Yes	Yes	Left thigh	Angiolipoma	Cured	True positive
6	Yes	Yes	Left fibular head	PMT	Cured	True positive
7	Yes	No	T_8_ vertebra	PMT	Cured	False negative
8	Yes	Yes	Left acetabulum	PMT	Cured	True positive
9	No	No	None identified	NA	Persistent disease	True negative
10	Yes	No	Calvarium	Sclerotic bone	Persistent disease	True negative
10	Yes	Yes	Right distal femur	No tumor	Persistent disease	False positive
11	Yes	Yes	Left greater trochanter	PMT	Cured	True positive
12	Yes	Yes	Left distal femur	Osteoma	Persistent disease	False positive
13	Yes	Yes	Left popliteal fossa	PMT	Cured	True positive
14	Yes	Yes	Left frontal lobe/meninges	PMT	Cured	True positive

NA = not applicable; PMT = phosphaturic mesenchymal tumor.

There were no complications or adverse events related to the selective venous sampling procedure.

## Discussion

The definitive treatment of TIO relies on identifying and resecting the causative tumor. However, in most cases, these are small, slowly growing mesenchymal neoplasms that are often difficult to detect with standard noninvasive radiologic studies, resulting in delayed diagnosis. In this study, we demonstrate the utility of selective venous sampling in the management of TIO. In our series of 14 consecutive patients with a definite diagnosis of TIO, we found this procedure particularly useful in specific circumstances. It helped to discriminate the culprit lesion from other suspicious lesions seen on imaging studies and thus potentially minimized the number of unnecessary resection procedures and rate of complications (eg, Fig. 2). In addition, it was shown to enhance the level of preoperative certainty prior to proceeding to technically challenging surgical procedures (eg, Fig. 1). In this case, the lesion identified on functional and anatomic imaging was in the fat pad of the heel of the foot. Reconstruction of the heel fat pad to maintain functionality involved a 10-hour operative repair that required transfer of a vascularized muscle flap from the arm. Findings from the selective venous sampling procedure added additional certainty and comfort in proceeding to this difficult operation. Another circumstance in which selective venous sampling is of utility is in distinguishing potentially false- from true-positive lesions. An example of this is shown in Fig. 4. In this case, the lesion was in the skull, which is a FDG-PET “blind spot” given the diffuse brain uptake. And while the pentetreotide scan clearly identified a lesion, the anatomic imaging study was more suggestive of a meningioma, which also takes up pentetreotide. In this case, we were able to access the far anterosuperior sagittal sinus and demonstrate a step-up in the plasma FGF-23 gradient, confirming that the pentetreotide study represented a true-positive study.

However, for studies in which there were no lesions on functional or anatomic imaging to guide the venous sampling (“blind studies”), sampling did not prove to be useful. There was one false-negative venous sampling procedure in this series (Fig. 3). This was a lesion in the T_8_ vertebral body. Because the lesion was identified on PET/CT, pentetreotide scan, MRI, and CT, this subject was included initially in the positive control group, with the expectation that the venous sampling would be positive. The most likely explanation for the fact that this study was negative is that the venous drainage from the vertebral skeleton is diffuse and the tumor venous effluent was dispersed over multiple veins. This area exhibits a unique anatomy, where the valve-less paraspinal veins of Batson's plexus exhibit bidirectional flow and communicate with the azygous vein system as well as the sacral and pelvic venous plexi. Studies performed for lesions in this anatomic location should be undertaken with consideration of this unusual venous anatomy.

The hope had been that we would be able to identify tumors in which there were no “leads” on functional or anatomic imaging. Unfortunately, this was not the case. This may be related to the relatively long half-life of intact FGF-23 (58 ± 34 minutes).(18) When a tissue secretes a factor with a relatively long half-life, the sampling must take place relatively close to the tumor, and it is likely in the cases categorized here as true negatives that we were not able to get the catheter tip close enough to the tumor. That said, while we have largely abandoned “blind studies,” in the case of patients with severe disease unable to be controlled medically, including the addition of cinacalcet to the regimen,(19) we most likely still would attempt a blind study as a last resort. An alternative explanation for negative studies is that there are cases of TIO that are not due to tumoral FGF-23 secretion. These cases may be due to nontumoral, diffuse secretion of FGF-23. In 2 subjects in whom we have not been able to identify an offending tumor despite multiple rounds of imaging, there is a suggestion of a diffuse bone marrow/hematopoietic abnormality. One subject carries the concomitant diagnosis of myelofibrosis, and the second has received a bone marrow transplant for aplastic anemia. While speculative, given that bone cells are the physiologic source of FGF-23 and ultimately derive from bone marrow stromal cells in adult life, these cases may represent instances in which bone marrow cells derived from bone marrow stromal cells pathologically secrete FGF-23 in a dysregulated/unregulated fashion.

Other considerations for negative studies include inaccessibility (or at least not readily accessible locations), such as the pulmonary venous tree, which would require a transcardiac arterial access approach. This may have been the case in a patient with TIO owing to pulmonary metastases who had a negative venous sampling study in another institution that we reported recently.(20)

This study builds on previous reports of the use of more limited venous sampling. For example, comparison of FGF-23 levels collected from superficial veins by simple venipuncture in patients with TIO and in whom the physical examination and imaging were highly suggestive of a culprit tumor has been performed.(14) The first report was of use of the procedure to confirm that a clinically palpable and clearly imaged right inguinal mass represented the FGF-23-secreting tumor.(12) In another case, venous sampling performed on a patient with multiple prior unrevealing CT and MRI studies revealed an approximately 70% increase in the FGF-23 concentration in the left femoral vein compared with a peripheral circulation FGF-23 level. In this case, a pentetreotide scan performed after the venous sampling procedure and repeat MRI restricted to the left knee clearly identified the tumor in this location.(15) In another case, a patient with a negative FDG-PET but an MRI study that revealed a tumor in the left greater trochanter underwent selective venous sampling of both lower extremity veins. A twofold step-up was noted in the left common iliac vein.(16) In yet another case, two-staged venous sampling was performed in a patient in whom pentetreotide and PET scanning and total-body CT and MRI failed to provide a suspect lesion. The first sampling procedure included all major veins and first branches and was negative for a diagnostic rise in any specific vein. Based on a difference in the mean values between the chest and abdominal body compartments, a second more detailed sampling was performed in the abdominal area focusing on smaller-vein tributaries. This suggested a right pelvic tumor location. Initially, the right ovary was removed but did not contain the FGF-23-secreting tumor. Eventually, a small right anterior uterine wall subserosal mass that in retrospect had been present on an earlier MRI was found to be the responsible tumor.(13) In all the reports cited earlier, though, imaging studies were able to detect the tumors. Some of these case reports originate from Japan, where diagnostic pentetreotide scintigraphy is not available. As in our series, with the exception of the subject with the frontal lobe lesion,(13) in all previously reported cases, tumors were identified on functional imaging and later confirmed with anatomic imaging. This has led us to take the following approach to the location of tumors causing TIO: First, we perform functional imaging (ie, PET/CT and pentetreotide/CT), followed by confirmation with high-resolution anatomic imaging (ie, CT and/or MRI); if discrimination is needed between multiple suspect lesions indentified on functional imaging or if greater certainty is needed that the identified lesion is in fact the FGF-23-secreting tumor, selective venous sampling and/or aspiration/biopsy with determination of the FGF-23 concentration in the washings is performed.

In conclusion, selective venous sampling for FGF-23 is a useful tool in the diagnosis of patients with TIO. It is especially helpful in distinguishing which site is the FGF-23-secreting tumor when multiple sites are identified on imaging studies. It is also useful when a high degree of certainty is needed preoperatively that the proposed site is the tumor. It does not appear to be useful in the absence of a suspicious lesion on imaging studies.
